# Shortened telomere length, anxiety and psychological stress in systemic lupus erythematosus: a cross-sectional study

**DOI:** 10.1186/s42358-025-00510-2

**Published:** 2025-12-17

**Authors:** Anna Carolina Faria Moreira Gomes Tavares, Carolina Ruas Freire Santos, Jéssica Martins Amaral, Ivonne Carolina Bolaños Burgos, Daniela Valadão Freitas Rosa, Marco Aurélio Romano-Silva, Maria Aparecida Camargos Bicalho, Gilda Aparecida Ferreira, Adriana Maria Kakehasi

**Affiliations:** 1 Post Graduation Program in Sciences Applied to Adult Health Care, Federal University of Minas Gerais (UFMG), Belo Horizonte, Brazil; 2 Rheumatology Service, University Hospital, Federal University of Minas Gerais (UFMG), Belo Horizonte, Minas Gerais Brazil; 3 Neurotec R National Institute of Science and Technology (INCT-Neurotec R), Federal University of Minas Gerais (UFMG), Belo Horizonte, Minas Gerais Brazil; 4 Departament of Clinical Medicine, Faculty of Medicine, Federal University of Minas Gerais (UFMG), Belo Horizonte, Minas Gerais Brazil

**Keywords:** Systemic lupus erythematosus, Telomere, Anxiety, Psychological stresses, Senescence

## Abstract

**Background:**

Oxidative stress and inflammation contribute to telomere length (TL) attrition, a hallmark of cellular senescence. Systemic lupus erythematosus (SLE) is associated with premature cellular aging; however, the clinical relevance of TL shortening remains unclear. This study aimed to compare the relative TL of SLE patients and healthy controls and explore its association with clinical features and disease burden.

**Methods:**

We conducted a cross-sectional study including adult SLE patients and age- and sex-matched healthy controls (18–60 years). Demographic and clinical data were collected, and TL was measured using quantitative polymerase chain reaction (qPCR). Among SLE patients, disease activity, cumulative damage, fatigue, depression, anxiety, stress, and physical activity were also assessed. Statistical analyses included descriptive statistics, Student’s t-test or Mann-Whitney test for group comparisons, Spearman’s correlation, and multiple linear regression models selected based on the lowest Akaike information criterion (AIC).

**Results:**

Sixty SLE patients (37 years ± 11.5) and 55 controls (38 years ± 10.5) were enrolled. SLE patients exhibited significantly shorter TL [median (IQR): 0.80 (0.29–2.94)] compared to controls [1.07 (0.38–2.32); *p* = 0.005]. In multivariate analysis, moderate anxiety was associated with shorter TL compared to none/low anxiety [β= -0.353 (95%CI: -0.645; -0.061); *p* = 0.019]. An alternative model indicated that moderate stress was also associated with reduced TL [β= -0.411 (95%CI: -0.771; -0.050); *p* = 0.026]. No significant associations were found between TL and disease activity or cumulative damage.

**Conclusions:**

SLE patients presented significantly shorter telomeres than healthy controls, with anxiety and stress symptoms contributing to TL attrition. Longitudinal studies are warranted to elucidate the clinical implications of TL shortening in SLE.

**Supplementary Information:**

The online version contains supplementary material available at 10.1186/s42358-025-00510-2.

## Background

Systemic lupus erythematosus (SLE) is a chronic autoimmune inflammatory disease characterized by hyperactivation of B cells, immune complex deposition, pathogenic autoantibody production, and multi-organ damage [1]. Emerging evidence suggests that accelerated immunosenescence — the premature aging of the immune system — plays a crucial role in SLE pathogenesis and progression, creating a cycle of inflammation, cellular damage, and increased morbidity [2].

The intersection between SLE and cellular senescence manifests through several shared immunological alterations. Patients with SLE exhibit significant changes in their immune cell repertoire, including decreased naïve T cells and oligoclonal expansion of memory T cells, mirroring age-associated immune dysregulation [2]. Additionally, SLE patients demonstrate an increased subpopulation of senescent T lymphocytes (CD4 + CD28-), which correlates with ocular, renal, vascular damage, and early gonadal failure [3]. This suggests that cellular senescence may contribute to organ-specific manifestations of the disease and related comorbidities.

At the molecular level, SLE-related senescence is characterized by enhanced cellular turnover, increased reactive oxygen species production due to mitochondrial dysfunction, and elevated pro-inflammatory cytokine secretion [4]. This persistent pro-inflammatory state creates a self-perpetuating cycle that further accelerates cellular aging through multiple pathways: inflammation increases cellular turnover, impairs telomerase activity, and induces oxidative damage to telomeric DNA, all of which contributing to telomere shortening [5]. This chronic inflammation-driven telomere attrition may explain why SLE patients face a heightened risk of cardiovascular disease, malignancies, and infectious complications later in life, mirroring conditions typically associated with advanced age.

Furthermore, this inflammatory burden extends to the central nervous system, where it can contribute to neuropsychiatric manifestations of SLE, including mood disorders and depression. The bidirectional relationship between inflammation and psychological distress appears particularly relevant in SLE, where patients with more severe inflammatory profiles often experience greater psychological burden, potentially exacerbating telomere shortening through both biological and behavioral pathways [6].

Telomeres—protein-DNA complexes consisting of highly conserved repetitive DNA sequences (TTAGGG)n at chromosome ends—serve as critical guardians of genomic stability and function as biomarkers of cellular senescence [5, 7]. Multiple factors influence telomere length (TL), including genetics, age, oxidative stress, inflammation, immune responses, lifestyle factors (smoking, alcohol consumption, obesity), and psychological conditions [4–6].

Consistent telomere shortening has been documented in SLE patients compared to healthy controls [1, 8–10]. A meta-analysis by Lee et al. confirmed significant differences in TL between SLE patients and controls [1]. Further research by Bridges et al. demonstrated shorter telomeres in individuals with longer disease duration when comparing childhood-onset and adult-onset SLE [8]. While Haque et al. found correlations between shortened TL and corticosteroid use and higher body mass index (BMI) in SLE patients, they did not observe associations between TL and disease activity or damage [9].

This study aims to compare relative TL between SLE patients and healthy controls while examining associations between TL and clinical parameters in SLE, including inflammatory markers, organ damage, and psychological symptoms. We hypothesize that the chronic inflammatory burden in SLE leads to progressive telomere shortening, which may serve as a measurable biomarker of cumulative disease impact and a predictor of age-related comorbidities. These findings may enhance our understanding of the mechanisms linking inflammation, cellular aging, and clinical outcomes in SLE, offering new insights into disease progression, risk stratification, and therapeutic monitoring.

## Materials and methods

### Participants

In this cross-sectional study, patients with SLE were randomly selected and matched with controls based on age and sex. Eligible participants were between 18 and 60 years old. All SLE patients met the 2019 classification criteria established by the European League Against Rheumatism (EULAR) and the American College of Rheumatology (ACR) [11]. Control participants were relatives of the patients without chronic immune-mediated inflammatory diseases. Recruitment was conducted consecutively from February to October 2022.

Exclusion criteria included the presence of acute or chronic infections, pregnancy or lactation, renal replacement therapy, cirrhosis, organ transplantation, non-rheumatic immune-mediated inflammatory diseases, structural damage to the central nervous system (CNS), and overlap syndromes, excluding antiphospholipid syndrome (APS) and secondary Sjögren’s syndrome.

Clinical and demographic information was obtained through structured interviews and medical record reviews during a single study visit. All data were securely stored in the REDCap database (Research Electronic Data Capture, https://project-redcap.org). Participants self-identified their ethnoracial classification and educational level based on the standards of the Brazilian Institute of Geography and Statistics [12]. Whole blood samples were collected from all participants.

The study protocol was approved by the Ethics and Research Committee of the Federal University of Minas Gerais (approval number: 50147321.9.0000.5149; August 21, 2021). All participants provided written informed consent. The study was conducted following the principles outlined in the Declaration of Helsinki, as revised in 2008.

### Telomere length analysis

Genomic DNA was extracted from peripheral blood samples using the saline method described by Lahiri and Numberger [13], with modifications by Cavalli et al. and Salazar et al. [14]. DNA quality and quantity were assessed using NanoDrop™ 2000/2000c spectrophotometer (ThermoScientific^®^, Waltham, MA, USA). Samples with 260/280 ratios between 1.8 and 2.0 were considered acceptable. DNA samples were standardized to a final concentration of 75 ng/20 µL and analyzed in triplicate.

Relative telomere length was measured using quantitative Real-Time Polymerase Chain Reaction (qPCR) on a 7500 Real-Time PCR System (Applied Biosystems^®^), following the method described by Cawthon [15]. This technique determines the ratio (T/S) between telomere repeat copy number (T) and single copy gene number (S). The average cycle thresholds (Cts) represent the number of PCR cycles required for the fluorescence signal to exceed the threshold level. Reactions were performed in triplicate, and samples with a coefficient of variation >2% were repeated. Data analysis was performed using Bio-Rad CFX Manager 3.1 software.

### Clinical assessment

All participants underwent assessment of demographic characteristics, anthropometric measurements, comorbidities, and history of malignancy. For patients with SLE, additional data were collected, including cumulative doses of antimalarials, glucocorticoids, and cyclophosphamide; measures of disease activity and cumulative damage; history of nephritis, neuropsychiatric lupus (NPSLE), APS, and severe or opportunistic infections (OIs) during follow-up; as well as evaluations of psychological status, fatigue, and physical activity levels.

### Disease activity and damage

Disease activity was evaluated using the modified systemic lupus erythematosus disease activity index 2000 (SLEDAI-2 K) [16], while cumulative organ damage was assessed with the Systemic Lupus International Collaborating Clinics/American College of Rheumatology Damage Index (SLICC/ACR-DI) [17].

Remission was defined based on the Definition of Remission in SLE (DORIS) criteria [18], and low disease activity was determined using the Lupus Low Disease Activity State (LLDAS) criteria [19]. Persistently active disease was characterized by a sustained SLEDAI-2 K score greater than 4 across the two most recent visits. A disease flare was defined as a 3-point or greater increase in the SLEDAI-2 K score at baseline compared to the previous assessment [20].

### Assessment of depression, anxiety, and stress

SLE patients were assessed for the presence of depression, anxiety, and stress symptoms using the Depression, Anxiety and Stress Scale-21 (DASS-21) [21]. The DASS-21 is a self-report instrument composed of three subscales, each scored using a four-point Likert scale (0, 1, 2, and 3), ranging from 0 (“did not apply to me at all”) to 3 (“applied to me most of the time”). Each subscale consists of seven items designed to evaluate one of the three domains.

Patients are classified according to symptom severity into the following categories: normal, mild, moderate, severe, and extremely severe. For multivariate analysis, the “severe” and “extremely severe” categories were grouped due to their clinical similarity.

### Assessment of fatigue

Fatigue was assessed using the Fatigue Subscale (FACIT-Fatigue) of the Functional Assessment of Chronic Illness Therapy (FACIT) score [22], which has been validated in populations with SLE. Total scores range from 0 to 52, with higher scores indicating lower fatigue levels.

### Assessment of physical activity

All patients completed the short version of the International Physical Activity Questionnaire (IPAQ) [23], designed for use in adults aged 18 to 65 years and based on self-reported physical activity during the previous seven days. The short form consists of nine items and addresses walking, moderate, and vigorous intensity activities, and sitting time during the past week. Patients were classified as sedentary, irregularly active, active and very active.

### Sample size

The sample size calculation was based on a previous study also evaluating telomere length in patients with SLE and healthy controls [6]. It was designed for two independent study groups, with a continuous variable as the primary endpoint. The statistical parameters used included a mean telomere length of 4.38 ± 3.58 for the study group (Group 1) and a mean of 6.37 for the comparison group (Group 2), with a significance level (α) of 5% and power (β) of 80%. This resulted in a required sample size of 51 participants per group.

### Statistical analysis

Results were expressed as frequencies and percentages for qualitative variables and mean standard deviation or median maximum and minimum for quantitative ones. Comparisons of relative TL and variables were performed using the t-Student and the Mann-Whitney tests. Normality was assessed using the Shapiro-Wilk test and homoscedasticity, the Levene test. Comparisons between sexes and groups were performed using the two-proportion test.

Three multiple regression analyses were performed, including TL as the dependent variable and, as independent variables, those with *p* ≤ 0.2 in the univariate analysis. The first considered the whole study population, and the second and third ones only the SLE patient group.

The degree of depression, anxiety, and stress, as well as physical activity, were categorized and grouped for these analyses. Spearman’s correlation coefficient evaluated correlations. Analyses were performed using R version 3.6.3 (R Foundation for Statistical Computing, Austria) and MINITAB programs (Minitab LLC, United States of America).

## Results

### Sample characteristics

The study comprised 60 patients with SLE and 55 control participants. Among those with SLE, 52 (86.7%) were female, with a mean age (SD) of 37 years (± 11.5) and an average disease duration of 12 years (± 5.8). In the control group, 48 individuals (87.3%) were female, with a mean age of 38 years (± 10.5). There were no significant differences between the groups considering sex (*p* = 0.923) or age (*p* = 0.737). However, the distribution of comorbidities varied significantly (*p* < 0.005): 35 SLE patients (56.7%) had more than two comorbidities, while 49 controls (89.1%) had none (Table 1).

**Table 1:** Demographic and clinical characteristics of 60 patients with systemic lupus erythematosus and 55 controls

Characteristics	Cases (*n* = 60)	Controls (*n* = 55)	*p*-value
**Sex**, ***n*(%)**			0.924*
*Female*	52 (86.7)	48(87.3)	-
**Age (years)**,** mean (SD) / median (min–max)**	37 (11.5) / 35 (19–60)	38 (10,5) / 37 (18–60)	0.737**
**Ethnic-racial classification**,** n (%)**			0.026*
* White*	17 (28.3)	10 (18.2)	-
* Black*	14 (23.3)	9 (16.4)	-
* Brown*	28 (46.7)	29 (52.7)	-
Others***	1 (1.7)	7 (12.7)	-
**Schooling (years)**,** n (%)**			0.855*
* 1–3*	3 (5)	0 (0)	-
* 4–7*	8 (13.3)	11 (20.4)	-
* 8–10*	5 (8.4)	6 (11.1)	-
* 11–14*	36 (60)	24 (44.4)	-
* >15*	8 (13.3)	13 (24.1)	-
**Body weight**,** mean (SD) / median (min-max)**	73.04 (17.19) / 69 (44–139)	71.29(14.00) / 72(40–105)	0.552**
**BMI**,** Kg/m**^**2**^	28.03 (6.31) / 28 (17–51)	27.48 (5.36) / 28 (17–46)	0.620**
**Comorbidities**,** n (%)**			< 0.005*
* None*	25 (41.7)	49 (89.1)	-
* 1–2*	35 (58.3)	6 (10.6)	-
* ≥ 3*	3 (5)	0 (0)	-
**Comorbidities**,** n (%)**			
* Obesity*	18 (30)	15 (27.3)	-
* Hypertension*	13 (21.7)	5 (9.1)	-
* Dyslipidemia*	11 (18.3)	1 (1.8)	-
* Diabetes mellitus*	6 (10)	2 (3.6)	-
* Smoking*	6 (10)	3 (5.5)	-
* COPD*	3 (5)	0 (0)	-
* CKD*	2 (3.3)	0 (0)	-
* Alcoholism*	1 (1.7)	0 (0)	-
Neoplasm****	1 (1.7)	0 (0)	-
**TL¹**,** mean**,** median**	0.91 (0.51) / 0.80 (0.29–2.94)	1.10 (0.45) / 1.07 (0.38–2.32)	**0.005** ^*^

Table data are presented as n (%), mean (SD), and median (minimum and maximum values). BMI: body mass index; CKD: chronic kidney disease; cm: centimeters; COPD: chronic lung disease; kg: kilograms; m^2^: meter squared; SD: standard deviation; TL: telomere length

*U of Mann-Whitney; **T-Test; ***Indigenous and yellows; ****Carcinoma in situ of the vulva

¹Adjusted for comorbidities and ethnic-racial classification

Regarding disease status, 39 patients (65%) were in remission or had low disease activity (LDA), eight (13.3%) experienced persistent activity, and 12 (20%) presented with active nephritis. Over the follow-up period, 27 patients (45%) had severe or opportunistic infections, and 16 (26.7%) required hospitalization.

Mental health assessments revealed that 25 patients (41.6%) showed moderate to very severe symptoms of depression, 27 (45%) experienced moderate to very severe anxiety, and 33 (55%) reported varying levels of stress, from mild to very severe. According to the IPAQ-SF, 45 patients (75%) were classified as physically active (Table 2).

**Table 2:** Clinical characteristics, fatigue, depression, anxiety and stress, and physical activity of 60 patients with systemic lupus erythematosus

**Characteristics**	
Diagnosis	
Disease duration*,* mean (SD) / median (min-max)*	12 (5.8) / 11 (1–27)
Age at diagnosis*,* mean (SD) / median (min-max)*	25 (10.1) / 22 (6–53)
**APS**,** n (%)**	9 (15)
**Infections**,** n (%)**	27 (45)
* Number of infections / patient*,* mean (SD) / median (min-max)*	0.78 (1.26) / 0 (0–6)
* Hospitalizations*	16 (26.7)
**Previous history (organ / system involvement)**,** n (%)**	
* Mucocutaneous*	50 (83.3)
* Joint*	49 (81.7)
* Cardiopulmonar*	18 (30)
* Nephritis*	31 (51.7)
* NPSLE*	14 (23.3)
* Hematological*	48 (80)
* Cutaneous vasculitis*	17 (28.3)
**Current history (organ / system involvement)**,** n (%)**	
* Mucocutaneous*	5 (8.3)
* Joint*	5 (8.3)
* Cardiopulmonar*	7 (11.7)
* Nephritis*	12 (20)
* NPSLE*	0 (0)
* Hematological*	7 (11.7)
* Cutaneous vasculitis*	0 (0)
**Disease activity**	
* Clinical remission*,* n (%)*	35 (58.3)
* LDA*,* n (%)*	4 (6.7%)
* Exacerbation*,* n (%)*	13 (21.7)
* Chronic activity*,* n (%)*	8 (13.3)
* SLEDAI-2 K*,* mean (SD) / median (min-max)*	
* At diagnosis*	11 (6.1) / 10 (2–25)
*In baseline*	4 (4.14) / 2 (0–18)
* PGA*	0.25 (0.55) / 0 (0–2)
**SDI**,** mean (SD) / median (min-max)**	1 (1.55) / 1 (0–6)
C3^***^, **mean (SD) / median (min-max)**	104 (30) / 106 (31–172)
C4^***^, **mean (SD) / median (min-max)**	20 (12) / 17 (2–54)
**Anti-native DNA positivity**,** n (%)**	31 (51.7)
**Previous treatment**,** n (%)**	
* Antimalarials*	60 (100)
* Prednisone*	60 (100)
* Azathioprine*	44 (73.3)
* Cyclophosphamide*	42 (70)
* Methylprednisolone*	36 (60)
* Metothrexate*	29 (48.3)
* Cyclosporine*	19 (31.7)
* Mycophenolate*	16 (26.7)
* Rituximab*	7 (11.7)
* Intravenous Human Immunoglobulin*	6 (10)
* Dapsone*	4 (6.7)
* Leflunomide*	3 (5)
* Thalidomide*	1 (1.7)
* Belimumab*	1 (1.7)
**Current treatment**,** n (%)**	
* Antimalarials*	45 (75)
* Prednisone*	
* ≤ 7*,*5 mg*	17 (28.3)
* > 7*,*5 mg*	11 (18.3)
* Azathioprine*	12 (20)
* Mycophenolate*	12 (20)
* Cyclosporine*	11 (18.3)
* Metothrexate*	11 (18.3)
* Rituximab*	4 (6.7)
* Cyclophosphamide*	3 (5)
* Dapsone*	1 (1.7)
* Leflunomide*	1 (1.7)
* Thalidomide*	1 (1.7)
* None*	2 (3.3)
* Stable immunosuppressant (6 months)*	36 (60)
**Accumulated doses**,** mean (SD) / median (min-max)**	
Antimalarials****	1,266,205 (1,649,784) / 990,737 (244,626 − 12,778,589)
Corticosteroids****	44,809 (33,516.47) / 37,088 (3,600 − 202,522)
Cyclophosphamide****	6,216 (5618.68) / 6,600 (800 − 23,600)
**FACIT – Fatigue**,** mean (DP)**,** median (min-max)**	36 (12.47) / 38 (2–52)
**DASS21**,** mean (SD)**,** median (min-max)**	42 (34.18) / 36 (0–42)
***Depression***,*** mean (SD)***,*** median (min-max)***	15 (14) / 12 (0–42)
* Normal / Mild*,* n (%)*	35 (58.3)
* Moderate*,* n (%)*	8 (13.3)
* Severe / very severe*,* n (%)*	17 (28.4)
***Anxiety***,*** mean (SD)***,*** median (min-max)***	11 (10.9) / 8 (0–40)
* Normal / Mild*,* n (%)*	33 (55)
* Moderate*,* n (%)*	12 (20)
* Severe / very severe*,* n (%)*	15 (25)
***Stress***,*** mean (SD)***,*** median (min-max)***	16 (12.2) / 14 (0–42)
* Normal / Mild*,* n (%)*	39 (65)
* Moderate*,* n (%)*	7 (11.7)
* Severe / very severe*,* n (%)*	14 (23.3)
**Physical activity**,** n (%)**	
* Very active*	4 (6.7%)
* Irregularly active*	8 (13.3%)
* Sedentary*	3 (5%)

Table data are presented as n (%), mean (SD), and median (minimum and maximum values). APS: antiphospholipid syndrome; dl: deciliter DVT: deep venous thrombosis; LDA: low disease activity; mg: milligrams; NPSLE: neuropsychiatric systemic lupus erythematosus; PGA: Physician Global Assessment; PTE: pulmonary thromboembolism; SD: standard deviation; SLEDAI-2 K: Systemic Lupus Erythematosus Disease Activity Index – 2000; SDI: Systemic Lupus International Collaborating Clinics – Damage Index

*In years; **in months; ***in mg/dl; ****in mg

### Telomere length analysis

SLE patients exhibited significantly reduced relative TL compared to healthy controls (median TL: 0.80 vs. 1.07, respectively; mean difference: -0.27; 95% CI: -0.38 to -0.07; *p* = 0.005) (Table 1).

Two univariate analyses were conducted using “relative TL” as the dependent variable. The first included the full sample (Table 1, supplementary material), and the second focused exclusively on the SLE cohort (Table 2, supplementary material). One extreme outlier (TL = 2.94) was identified and excluded through residual analysis, as seen in Fig. 1 (adjusted model residuals plot, *n* = 114), and in Fig. 2 (adjusted model residuals plot, *n* = 59), in the supplementary material.

The initial multivariate model assessed the combined effects of disease status (SLE vs. control), ethnoracial classification, and presence of comorbidities on relative TL across the entire study population (*n* = 114). After adjusting for these variables, SLE patients continued to exhibit significantly shorter TL compared to controls (mean difference: -0.264; 95% CI: -0.432 to -0.096; *p* = 0.002) (Table 3, supplementary material).

**Table 3:** Second multiple regression model, anxiety (chosen model, *n* = 59)

Characteristics	Adjustments				
	Initial model			Final model	
	Coeficient (CI_95%_)	*p*		Coeficient (CI_95%_)	*p*
**Time of diagnosis (years)**	0.013 (-0.008; 0.033)	0.217		–	–
**Presence of infections**	-0.145 (-0.384; 0.093)	0.227		–	–
**NPSLE**	-0.215 (-0.527; 0.097)	0.172		–	–
**Antimalarials (ac. dose)**	-5.01E-08 (-1.18E-07; 1.82E-08)	0.147		–	–
**Anxiety**					
* Moderate*	-0.111 (-0.449; 0.227)	0.513		-0.353 (-0.645; -0.061)	**0.019**
* Severe or very severe*	0.171 (-0.162; 0.505)	0.307		0.056 (-0.214; 0.326)	0.679
**Stress**					
* Moderate*	-0.160 (-0.584; 0.263)	0.450		–	–
* Severe or very severe*	0.006 (-0.348; 0.359)	0.973		–	–
**Physical activity**					
* Irregularly active*	-0.029 (-0.369; 0.311)	0.865		–	–
* Sedentary*	0.554 (0.001; 1.108)	0.050		–	–
**Adjusted R²**					11.5%

Ac: accumulated; CI: confidence interval; NPSLE: neuropsychiatric systemic lupus erythematosus

Variables such as clinical history, current treatments, disease activity and damage indices, psychiatric symptoms (depression, anxiety, and stress), and physical activity levels were incorporated into two multivariate regression models (Tables 4 and 5, supplementary material).

The multivariate models were developed, with model selection guided by multicollinearity assessments between psychological variables and by comparison of Akaike information criterion (AIC) values to determine optimal model fit (Table 4. Adjustment steps for the multiple regression models, *n* = 59, supplementary material). The selected model, which included anxiety levels, demonstrated that individuals with moderate anxiety had, on average, a relative TL that was 0.353 shorter (95% CI: -0.645 to -0.061) than those with mild or no anxiety symptoms (Table 3). An alternative model incorporating stress levels is presented in Table 5 (Third multiple regression model, stress - alternative model, *n* = 59), supplementary material.

## Discussion

Our study demonstrates that SLE patients have significantly shorter relative TL than controls without immune-mediated inflammatory diseases. This finding persisted after adjustment for comorbidities in multivariate analysis. These results align with previous studies [1, 6–8] and represent the first evaluation of TL in Brazilian SLE patients, including its association with mood disorders.

It is well established that individuals with SLE face a range of mental health challenges, arising either from the neuropsychiatric manifestations intrinsic to the disease or from the psychosocial stressors inherent to living with a chronic illness. Compared to the general population, patients with SLE exhibit a markedly higher prevalence of depression, anxiety, cognitive dysfunction, and other psychiatric conditions [24]. Emotional distress has been closely linked to systemic inflammation and oxidative stress, both of which are implicated in telomere attrition and the pathogenesis of SLE. Chronic psychological stress is known to accelerate biological aging, predominantly through inflammatory pathways [25].

In our sample, we observed a prevalence of anxiety symptoms in 27% of patients, stress symptoms in 21%, and depressive symptoms in 25%, findings that are consistent with previous reports by Quilter et al. and Zhang et al. [26, 27]. Our multiple regression analysis demonstrated a significant inverse association between anxiety levels and telomere length in SLE patients, aligning with findings reported in other populations [28]. Psychiatric disorders, particularly depression and anxiety, have profound effects on health outcomes in SLE, contributing to increased cardiovascular disease (CVD) risk and severely impairing quality of life [15]. Darrow and colleagues further substantiated the strong association between psychiatric conditions, including post-traumatic stress disorder (PTSD), depression, and anxiety, and telomere shortening [26].

Anxiety levels in our cohort were assessed using the DASS-21, a self-report instrument that correlates well with other standardized measures of emotional states, such as the Beck inventories [21]. The DASS-21 evaluates anxiety as a state characterized by fear and apprehension, marked by discomfort in anticipation of perceived threats [21]. Notably, stress and anxiety are intrinsically interconnected and often clinically indistinguishable.

The pathogenesis of SLE is multifactorial, involving genetic susceptibility, hormonal imbalances, environmental exposures, and psychological stressors. Increasing evidence highlights a bidirectional relationship between inflammatory processes and SLE-associated mood and anxiety disorders. Furthermore, depression may exacerbate the risk of CVD—one of the leading causes of mortality in SLE—through both biological mechanisms (e.g., elevated catecholamine levels and autonomic dysfunction) and behavioral pathways (e.g., impaired self-care behaviors) [29].

We did not observe a relationship between relative TL and disease activity, in contrast to the findings reported by Wu et al. [8], nor did we find an association with cumulative disease damage. Haque and colleagues [11] reported longer telomeres in patients who were current steroid users, smokers, or had higher BMI compared to healthy controls. However, in our study, no significant associations were found between TL and comorbidities or prior treatments.

We specifically evaluated the impact of antimalarials, glucocorticoids, and cyclophosphamide on relative TL, as these medications are commonly used in SLE management and have been linked to long-term organ damage, one of our clinical outcomes of interest. Additionally, no association was found between disease duration and relative TL in our cohort. This contrasts with the findings of Bridges et al. [9], who reported an inverse relationship between time since diagnosis and TL, particularly when comparing childhood-onset to adult-onset lupus.

Our study has limitations. Notably, the sample size was calculated based on TL comparisons between SLE patients and controls, and was not powered to detect associations with secondary outcomes, which may have influenced these findings. It is important to highlight that the sample size determination relied on previously published data comparing TL between cases and controls. Additionally, the cross-sectional nature of the study may have limited the ability to detect associations between TL, disease activity, and cumulative damage in SLE. Finally, anxiety and depression were not assessed in the control group. Therefore, this study cannot determine whether the association between telomere shortening and anxiety occurs independently of SLE.

Despite these limitations, the study also presents important strengths. The sample size exceeded the minimum required for the primary outcome analysis and included participants representative of Brazil’s genetically diverse population, enhancing the generalizability of the findings. Furthermore, the use of qPCR for TL measurement ensures methodological robustness. Compared to other techniques, qPCR is advantageous due to its simplicity, cost-effectiveness, and low DNA input requirement.

## Conclusions

This study confirms that patients with SLE exhibit shorter telomeres compared to healthy controls and suggests a potential association between anxiety and telomere shortening within this population. To better understand the clinical implications of telomere attrition, future longitudinal studies are warranted to assess its influence on disease activity, cumulative organ damage, and the onset of comorbidities such as cardiovascular disease, infections, and malignancies.

## Supplementary Information

Below is the link to the electronic supplementary material.

Supplementary Material 1

## Data Availability

All data generated or analyzed during this study are included in this published article and its supplementary information files.

## Abbreviations

Ac: Accumulated
ACR: American College of Rheumatology
AIC: Akaike information criterion
APS: Antiphospholipid syndrome
BMI: Body mass index
CD: Cluster of differentiation
CI: Confidence interval
CKD: Chronic kidney disease
cm: Centimeters
CNS: Central nervous system
COPD: Chronic lung disease
Cts: Cycle thresholds
CVD: Cardiovascular disease
DASS-21: Depression, anxiety and stress scale-21
DI: Damage index
dl: Deciliter
DNA: Deoxyribonucleic acid
DVT: Deep venous thrombosis
DORIS: Definition of remission in SLE
EULAR: European League Against Rheumatism
FACIT: Functional assessment of chronic illness therapy
IPAQ-SF: International physical activity questionnaire – short form
IQR: Interquartile range
kg: Kilograms
LDA: Low disease activity
LLDAS: Lupus low disease activity state
m^2^: Meter squared
mg: Milligrams
NPSLE: Neuropsychiatric lupus
PGA: Physician global assessment
PTE: Pulmonary thromboembolism
PTSD: Post-traumatic stress disorder
qPCR: Quantitative polymerase chain reaction
REDCap: Research electronic data capture
SD: Standard deviation
SLE: Systemic lupus erythematosus
SLEDAI-2K: Systemic lupus erythematosus disease activity index 2000
SLICC: Systemic lupus international collaborating clinics
PTE: Pulmonary thromboembolism
TL: Telomere length
m^2^: Meter squared
