# Supplementary material for: Shortened telomere length, anxiety and psychological stress in systemic lupus erythematosus: a cross-sectional study
Source: Adv Rheumatol. 2025 Dec 17;66:8. doi: 10.1186/s42358-025-00510-2 (PMC13488778; doi:10.1186/s42358-025-00510-2)
Supplement: Supplementary file 1 [file 42358_2025_510_MOESM1_ESM.docx]

Table 1 (supplementary table) – Univariate analysis for relative telomere length of whole sample (n = 114)

| **Characteristics** | **Coeficient** | **CI_95%_** |  | **p-value** |
| --- | --- | --- | --- | --- |
| **Patients** | -0.264 | -0.432; -0.096 |  | **0.002** |
| **Sex (female)** | -0.040 | -0.306; 0.227 |  | 0.769 |
| **Age (years)** | -0.002 | -0.010; 0.006 |  | 0.624 |
| **Ethnic-racial classification** |  |  |  |  |
| *White* | -0.059 | -0.156; 0.273 |  | 0.589 |
| *Black* | 0.089 | -0.142; 0.319 |  | 0.448 |
| *Others* | 0.414 | 0.067; 0.761 |  | **0.020** |
| **Schooling (years)** |  |  |  |  |
| *Zero to 3* | -0.163 | -0.064; 0.316 |  | 0.501 |
| *Four to 10* | -0.025 | -0.226; 0.175 |  | 0.802 |
| **BMI (kg/m^2^)** | 0.003 | -0.013; 0.018 |  | 0.740 |
| **Presence of comorbidities** | -0.231 | -0.406; -0.056 |  | **0.010** |
| **Comorbidities** |  |  |  |  |
| *Smoking* | -0.031 | -0.373; 0.312 |  | 0.860 |
| *Hypertension* | -0.068 | -0.307; 0.172 |  | 0.576 |
| *Diabetes mellitus* | -0.191 | -0.531; 0.150 |  | 0.270 |
| *Dyslipidemia* | 0.028 | -0.257; 0.313 |  | 0.847 |
| *Obesity* | 0.030 | -0.147; 0.207 |  | 0.740 |
| *CKD* | -0.041 | -0.979; 0.896 |  | 0.931 |
| *COPD* | -0.049 | -0.595; 0.497 |  | 0.859 |

BMI: body mass index; CI: confidence interval; CKD: chronic kidney disease; COPD: chronic obstructive pulmonary disease.

Table 2 (supplementary table). Univariate analysis for relative telomere length among SLE patients (n = 59)

| **Characteristics** | **Coeficient** | **CI_95%_** | **p-value** |
| --- | --- | --- | --- |
| **Sex (female)** | -0.007 | -0.373; 0.358 | 0.969 |
| **Age (years)** | 0.006 | -0.005; 0.016 | 0.274 |
| **Time since diagnosis (years)** | 0.017 | -0.003; 0.036 | **0.102** |
| **Age at diagnosis (years)** | 0.002 | -0.009; 0.014 | 0.691 |
| **BMI (kg/m^2^)** | 0.001 | -0.018; 0.021 | 0.889 |
| **Comorbidities** |  |  |  |
| *<2* | -0.042 | -0.294; 0.210 | 0.738 |
| *≥2* | -0.106 | -0.603; 0.391 | 0.671 |
| **Alcohol intake** | -0.313 | -1.225; 0.599 | 0.495 |
| **Smoking** | -0.094 | -0.518; 0.330 | 0.660 |
| **Presence of infections during follow-up** | -0.167 | -0.401; 0.067 | **0.158** |
| **Previous history** |  |  |  |
| *Nephritis* | -0.032 | -0.268; 0.204 | 0.786 |
| *NPSLE* | -0.190 | -0.463; 0.083 | **0.169** |
| **Treatment (accumulated)** |  |  |  |
| *Antimalarials (years of use)* | 0.010 | -0.011; 0.032 | 0.330 |
| *Antimalarials* | -4.68E-08 | -1.18E-07; 2.41E-08 | **0.191** |
| *Corticosteroids* | -1.04E-06 | -4.58E-06; 2.50E-06 | 0.560 |
| *Cyclophosphamide* | -1.02E-05 | -3.31E-05; 1.27E-05 | 0.376 |
| **Disease activity and damage** |  |  |  |
| *SLEDAI-2K* – *diagnosis* | 0.006 | -0.014; 0.026 | 0.532 |
| *SLEDAI-2K* – *baseline* | -0.007 | -0.035; 0.022 | 0.646 |
| *SDI* – *baseline* | 0.010 | -0.066; 0.086 | 0.786 |
| **Fatigue, depression, anxiety and stress** |  |  |  |
| *FACIT - Fatigue* | -0.006 | -0.015; 0.004 | 0.227 |
| *DASS21* | 3.06E-04 | -0.004; 0.003 | 0.861 |
| *Depression* | 3.66E-05 | -0.008; 0.009 | 0.993 |
| *Moderate* | -0.204 | -0.560; 0.152 | 0.257 |
| *Severe / very severe* | -0.020 | -0.289; 0.249 | 0.882 |
| *Anxiety* | 0.002 | -0.008; 0.013 | 0.650 |
| *Moderate* | -0.353 | -0.645; 0.061 | **0.019** |
| *Severe / very severe* | 0.056 | -0.214; 0.326 | 0.679 |
| *Stress* | -0.003 | -0.013; 0.007 | 0.552 |
| *Moderate* | -0.411 | -0.771; 0.050 | **0.026** |
| *Severe / very severe* | -0.078 | -0.352; 0.196 | 0.572 |
| **Physical activity** |  |  |  |
| *Irregularly active* | -0.161 | -0.499; 0.178 | 0.346 |
| *Sedentary* | 0.422 | -0.106; 0.950 | **0.115** |

BMI: body mass index; CI: confidence interval; DASS: Depression Anxiety Stress Scale; FACIT: Functional Assessment of Chronic Illness Therapy; kg: kilograms; m^2^: meter squared; NPSLE: neuropsychiatric systemic lupus erythematosus; PGA: Physician Global Assessment; PTE: pulmonary thromboembolism; SD: standard deviation; SLEDAI-2K: Systemic Lupus Erythematosus Disease Activity Index – 2000; SDI: Systemic Lupus International Collaborating Clinics – Damage Index.; SD: standard deviation.

Table 3 (supplementary table) – Multiple regression model for relative telomere length outcome (first model, n = 114)

| **Characteristics** | **Adjustment** | | | |
| --- | --- | --- | --- | --- |
|  | **Initial model** | | **Final model** | |
|  | **Coeficient (CI_95%_)** | **p** | **Coeficient (CI_95%_)** | **p** |
| **SLE patients** | -0.182 (-0.386; 0.023) | 0.081 | -0.264 (-0.432; -0.096) | **0.002** |
| **Ethnic-racial classification** |  |  |  |  |
| *White* | 0.084 (-0.126; 0.294) | 0.427 | – | – |
| *Black* | 0.097 (-0.129; 0.323) | 0.397 | – | – |
| *Others* | 0.330 (-0.012; 0.672) | 0.059 | – | – |
| **Comorbidities** | -0.116 (-0.322; 0.089) | 0.265 | – | – |
| **Adjusted R²** |  |  |  | 8,3 |

CI: confidence interval

Table 4 (supplementary table). Adjustment steps for the multiple regression models (n = 59)

| **Characteristics** | **p-value** | | | | | | | | | **Final model** |
| --- | --- | --- | --- | --- | --- | --- | --- | --- | --- | --- |
|  | **I** | **II** | **III** | **IV** | **V** | **VI** | **VII** | **VIII** | **IX** |  |
| **Time of diagnosis (years)** | 0.217 | 0.160 | 0.117 | 0.122 | X | X | X | X | X | X |
| **Presence of infections** | 0.227 | 0.205 | 0.158 | X | X | X | X | X | X | X |
| **NPSLE** | 0.172 | 0.092 | 0.072 | 0.080 | 0.058 | 0.085 | X | X | X | X |
| **Antimalarials (ac. dose)** | 0.147 | 0.132 | 0.073 | 0.085 | 0.104 | X | X | X | X | X |
| **Anxiety** |  |  |  |  |  |  |  |  |  |  |
| *Moderate* | 0.513 | 0.373 | X | X | X | X | X | **0.019** | X | **0.019** |
| *Severe or very severe* | 0.307 | 0.246 | X | X | X | X | X | 0.679 | X | 0.679 |
| **Stress** |  |  |  |  |  |  |  |  |  |  |
| *Moderate* | 0.450 | X | X | X | X | X | X | X | **0.026** | X |
| *Severe or very severe* | 0.973 | X | X | X | X | X | X | X | 0.572 | X |
| **Physical activity** |  |  |  |  |  |  |  |  |  |  |
| *Irregularly active* | 0.865 | 0.837 | 0.847 | 0.695 | 0.438 | 0.497 | 0,346 | X | X | X |
| *Sedentary* | 0.050 | **0.034** | **0.031** | **0.030** | **0.041** | **0.049** | 0,115 | X | X | X |
| **Adjusted R^2^** |  |  |  |  |  |  |  | 11.5% | 8.5% | 11.5% |
| **AIC** |  |  |  |  |  |  |  | 72.9 | 74.8 | 72.9 |

Ac: accumulated; AIC: Akaike information criterion; NPSLE: neuropsychiatric systemic lupus erythematosus

Table 5 (supplementary table). Third multiple regression model, stress (alternative model, n = 59)

| **Characteristics** | **Adjustments** | | | | |
| --- | --- | --- | --- | --- | --- |
|  | **Initial model** | |  | **Alternative final model** | |
|  | **Coeficient (CI_95%_)** | **p** |  | **Coeficient (CI_95%_)** | **p** |
| **Time of diagnosis (years)** | 0.013 (-0.008; 0.033) | 0.217 |  | – | – |
| **Presence of infections** | -0.145 (-0.384; 0.093) | 0.227 |  | – | – |
| **NPSLE** | -0.215 (-0.527; 0.097) | 0.172 |  | – | – |
| **Antimalarials (ac. dose)** | -5.01E-08 (-1.18E-07; 1.82E-08) | 0.147 |  | – | – |
| **Anxiety** |  |  |  |  |  |
| *Moderate* | -0.111 (-0.449; 0.227) | 0.513 |  | – | – |
| *Severe or very severe* | 0.171 (-0.162; 0.505) | 0.307 |  | – | – |
| **Stress** |  |  |  |  |  |
| *Moderate* | -0.160 (-0.584; 0.263) | 0.450 |  | - 0.411 (-0.771; -0.050) | **0.026** |
| *Severe or very severe* | 0.006 (-0.348; 0.359) | 0.973 |  | -0.078 (-0.352; 0.196) | 0.572 |
| **Physical activity** |  |  |  |  |  |
| *Irregularly active* | -0.029 (-0.369; 0.311) | 0.865 |  | – | – |
| *Sedentary* | 0.554 (0.001; 1.108) | 0.050 |  | – | – |
| **Adjusted R²** |  |  |  |  | 8.5% |

Ac: accumulated; CI: confidence interval; NPSLE: neuropsychiatric systemic lupus erythematosus

Figure 1. Adjusted model residuals plot (n = 114), supplementary figure

**D**

**A**

**C**

**B**

A. Normal probability plot of the residuals; B. Residuals versus the fitted values; C. Histogram of the residuals; D. Residuals versus the order of the data.

Figure 2. Adjusted model residuals plot (n = 59), supplementary figure

**B**

**D**

**C**

**A**

A. Normal probability plot of the residuals; B. Residuals versus the fitted values; C. Histogram of the residuals; D. Residuals versus the order of the data.
